# Tamoxifen exposure in pregnancy after synchronous breast and thyroid cancer

**DOI:** 10.3332/ecancer.2020.1125

**Published:** 2020-10-15

**Authors:** Abeid M Athman Omar, Amany Abdel-Bary, Rasha O Elsaka

**Affiliations:** 1 Department of Clinical Oncology and Nuclear Medicine, Faculty of Medicine, Alexandria University, Champlion Street, Alazarita, Alexandria 21131, Egypt; 2 Department of Pathology, Faculty of Medicine, Alexandria University, Champlion Street, Alazarita, Alexandria 21131, Egypt; a https://orcid.org/0000-0003-4081-8547; b https://orcid.org/0000-0002-5393-8903; c https://orcid.org/0000-0002-5320-8302

**Keywords:** breast cancer, thyroid cancer, tamoxifen, pregnancy

## Abstract

Thyroid and breast cancer are the most common cancers among young women, which are either synchronous or metachronous, but the association is yet to be elucidated. With the improvement of diagnosis and treatment, there is an increase in breast and thyroid cancer survivors. Hence, attention is shifting towards survivorship. Here, we report the case of a young lady diagnosed with synchronous thyroid and breast cancer who unexpectedly became pregnant during tamoxifen treatment. After a multidisciplinary discussion, endocrine therapy was interrupted and she delivered a healthy baby at term. In conclusion, oncologists should be aware of breast and thyroid cancer co-occurrence and examinations should be conducted together in diagnosis and follow-up. Also, pregnancy is feasible and can be considered after synchronous breast and thyroid cancer diagnosis. Physicians need to emphasise the use of barrier contraceptives to patients undergoing endocrine therapy. However, the optimum timing for pregnancy after breast cancer and the safety of endocrine therapy interruption in hormonal-positive patients should be discussed and managed by a multidisciplinary team.

## Introduction

While breast cancer (BC) is the most common malignancy in women worldwide, thyroid cancer (TC) is the most prevalent endocrine cancer. Although the incidence of breast and thyroid cancer (BC–TC) continues to rise over the last few decades, in contrast, the mortality rate is declining [1, 2].

It is well known that BC-TC might co-exist either in a synchronous or metachronous pattern, although the former is rare. More patients with TC are prone to later developing BC than vice versa [3]. The majority of TC are well-differentiated and indolent with a good prognosis [4]. Since TC is more indolent compared to BC, the latter determines survival.

The aetiology for the association between BC and TC has not been fully elucidated. Survivors with a prior history of ionising radiation exposure have been reported to have an increased BC–TC incidence. Also, there is an interaction between oestrogen and thyroid hormones. The secretion of thyroid-stimulating hormone appears to be stimulated by oestrogen. Moreover, TC has been shown to highly express oestrogen receptors (ER). Synchronous BC–TC can occur in patients with Cowden syndrome (CS). However, some patients with synchronous disease lack phosphatase and tensin homolog gene (PTEN) mutation; it is known as the Cowden-like syndrome (CLS) [5–9].

BC is uncommon in women less than 40-year old. However, its incidence is on the rise, reaching up to 25% in developing countries [10]. The advances in diagnosis and treatment have led to a tremendous decline in the mortality rate, notably in young women, leading to more young BC survivors [11]. Noteworthy, the recent trend of delayed childbearing in women is associated with an increase in BC diagnosis and treatment before completing their family. Consequently, more BC survivors are now willing to become pregnant to complete childbearing [12, 13].

While there are few case reports or series in the literature on synchronous BC–TC, we did not find a case report of a woman becoming pregnant after primary synchronous BC–TC in our search in PubMed. Therefore, to our knowledge, this is the first case report to highlight the feasibility of pregnancy after synchronous BC–TC. Herein, we present a case of a young woman with synchronous BC–TC who unexpectedly become pregnant while on endocrine therapy (ET) and briefly discuss the available literature.

## The case

A 38-year-old female presented at Alexandria clinical oncology department in November 2015 with a right painless breast lump and a right neck mass, both of which had been present for more than one year. No concomitant disease was present. The physical examination revealed a firm, mobile, non-tender right breast mass without skin changes in the upper outer quadrant and palpable mobile axillary lymph nodes (ALN). The neck examination found an enlarged right thyroid lobe with a firm mass and without palpable cervical nodes. On imaging, the ultrasound and mammogram of the right breast showed a highly suspicious area of 2.1 × 2.2 cm at the axillary tail with multifocal lesions, the largest being 0.9 × 7.5 mm at 9 and 10 o’clock, and suspicious ipsilateral ALNs – BI-RADS 5. The left breast looked normal. The neck ultrasound showed a moderately enlarged right thyroid lobe with a heterogeneous solid and cystic mass of 3.8 cm × 2.0 cm with reactive bilateral level II cervical lymph nodes. The left thyroid lobe looked normal. Fine needle aspiration and core biopsy were carried out, which showed invasive ductal carcinoma (IDC) in the breast, while atypical follicular neoplasm with cystic changes was suggestive of papillary thyroid carcinoma in the thyroid*.* The CT scan of the neck, chest, abdomen and pelvis did not show any metastases.

**Figure 2.**
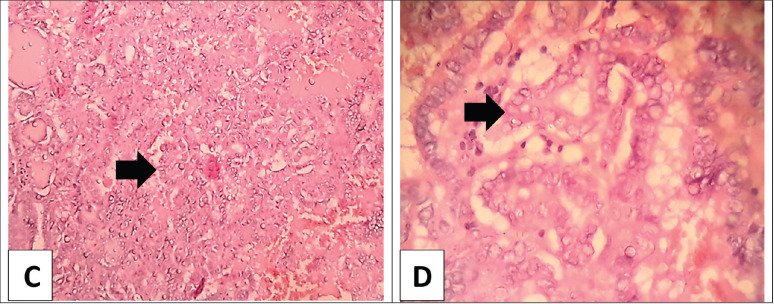
(C): Low power view of the thyroid papillary carcinoma; classic variant with multiple papillary structures having fibrovascular cores (arrow) separated by vascularized stroma (H&E × 100). (D): Higher power view of the thyroid papillary carcinoma; the papillae are lined with a layer of cells showing evident nuclear features with large ground glass overlapped nuclei (arrow) (H&E × 400).

In December 2015, the patient underwent a modified radical mastectomy (MRM). The final pathology report showed a multifocal, 5-cm, grade 3 IDC. Lymphovascular invasion and a small ductal carcinoma *in situ* component were present. Two out of 16 ALNs were involved with extracapsular extension; stage IIB BC (Figure 1). Immunohistochemistry stained strongly positive for oestrogen and progesterone receptors and was negative for human epidermal growth factor receptor-2 (HER-2). One month later, the patient underwent total thyroidectomy, and the pathology report revealed a 2.5cm × 2.5cm × 2cm papillary carcinoma. Focal vascular and extracapsular invasions were present (Figure 2).

She then started levothyroxine 100 mcg and adjuvant chemotherapy (5-FU, adriamycin, cyclophosphamide) FAC six cycles. She remained premenopausal post-chemotherapy. This was followed by adjuvant tamoxifen and chest wall irradiation, including the supraclavicular area (42 Gy in 16 fractions), was carried out. Radio-iodine ablation was planned but not carried out as it was unavailable at that time. The levothyroxine dose was adjusted over time, according to serial thyroid function measurements.

Four months after the completion of radiation therapy and during tamoxifen treatment, the patient experienced morning sickness. A pregnancy test turned out to be positive, and the Beta-human chorionic gonadotropin level (6, 3675 IU/ml) corresponded to a 7–8 weeks pregnancy. Tamoxifen was immediately stopped and the patient was referred to a gynaecologist. After multidisciplinary consultation, it was agreed to continue with the pregnancy and levothyroxine and to discontinue tamoxifen until the delivery. The pregnancy was uneventful and she delivered through a caesarean section a normal healthy baby at term. After birth, the patient decided not to breastfeed and resumed tamoxifen in August 2017. She was well until April 2020, when she developed a resectable chest wall recurrence. Her baby is well with expected developmental milestones. On the last follow-up, the patient was still alive.

## Discussion

Although synchronous BC–TC is rare compared to metachronous tumours, they share some clinical–pathological similarities. They tend to present at an early stage, with well to moderate-differentiation, small tumour size, negative or less positive lymph nodes and are common in premenopausal women [14]. Besides, patients often present with hormone receptor-positive BC [15, 16]. In this case report, the patient had a hormonal positive, stage IIB, but poorly differentiated IDC.

BC–TC also share oestrogen receptor α (ERα), oestrogen receptor β (ERβ) and HER-2 expression and phosphatidylinositol 3-kinase pathway (PI3K) pathway activation [17]. ERα increases the proliferation rate of cancer cells, while ERβ is pro-apoptotic. 17β-oestradiol (E2) is known to increase the activity of ERα while inhibiting ERβ. Oestradiol increases the proliferation of ER-positive TC in human and animal cell lines [8, 18]. Patients with BC–TC usually have ER-positive BC, while TC expresses ERs [19]. Our patient had strong ER-positive BC.

At the genomic level, BC–TC has been shown to have P53, reticular activating system gene, rearranged during transfection gene and PTEN mutations driving signals through PI3K and mitogen-activated protein kinase pathway pathways [20–24]. Synchronous BC–TC commonly occurs in Cowden and CLS. CS presents with synchronous thyroid, breast and endometrial cancers in which patients have PTEN mutation, while the CLS is associated with succinate dehydrogenase mutation and enhanced P53 degradation [9, 25]. This patient had only synchronous BC–TC; however, genetic/germline testing was not carried out in our patient.

Patients diagnosed with synchronous BC–TC can undergo simultaneous or sequential surgery [26, 27]. In a series of 12 patients who were diagnosed preoperatively with synchronous BC–TC, they all underwent simultaneous mastectomy and thyroidectomy. All patients had total thyroidectomy, while the BC was operated either by MRM or simple mastectomy, with either ALN or sentinel lymph node dissection. The only postoperative complications were seroma and infection in two separate patients. All the patients were still disease-free at the end of their follow-up period of 15.6 months [27]. This shows that simultaneous thyroidectomy and mastectomy are quite safe and feasible. However, longer follow-up and larger series are needed to provide substantial guidelines. In our case, similar to other series, the patient had sequential surgery—MRM followed by thyroidectomy.

The incidence of young BC survivors is on the rise. Furthermore, more women are shifting towards later childbearing and, as a result, are diagnosed with BC before completing their families [28, 29]. Tamoxifen is used routinely as an adjuvant endocrine treatment (ET) in hormone receptor-positive early BC. Animal and human studies have shown that tamoxifen and its metabolites also interact with embryonic and foetal tissues, which could lead to teratogenicity if used in pregnancy. A study by Braems et al. showed that the unexpected use of tamoxifen in pregnancy could cause 25%–33% adverse outcomes, including preterm labour, spontaneous abortion, stillbirth with foetal defects and congenital malformations, especially urogenital [30–32]. Children with perinatal exposure to tamoxifen may have a risk of developing reproductive toxicities, such as uterine anomalies, neoplasia or atrophy and precocious puberty. Therefore, tamoxifen should never be used in pregnancy or during the lactation period. Physicians are required to counsel and recommend the use of barrier contraceptives to women taking tamoxifen. This should include educating the patients about tamoxifen teratogenicity and foetal adverse effects. If inadvertent pregnancy occurs while on tamoxifen, it should be stopped immediately and the risks should be discussed. In case a patient wishes to become pregnant, at least a three-month tamoxifen wash-out period is required before one can safely become pregnant [32, 33]. To the best of authors’ knowledge, this is the first case report of a synchronous BC–TC survivor becoming pregnant, although unplanned. Despite being counselled on the use of non-hormonal contraceptives, the patient unexpectedly became pregnant with eight weeks of foetal exposure to tamoxifen; however, she delivered a healthy normal baby at term. Longer follow-up is required to rule out any long-term effects of *in utero* exposure to tamoxifen.

Pregnancy following BC is considered safe even in patients with ER-positive BC and is supported by international guidelines [34, 35]. Nevertheless, the optimal timing for safe pregnancy following BC diagnosis is unknown. Generally, 5 years from diagnosis is regarded as not detrimental, albeit without survival advantage than those becoming pregnant before that. In a nutshell, at least 2 years may be necessary before one can safely conceive, and full recovery of the ovarian function should be considered (as it varies with the patient’s age) [35, 36]. However, the safety of the temporary interruption of ET is not fully elucidated [34]. A large international trial (Pregnancy Outcome and Safety of Interrupting Therapy for women with endocrine responsive breast, POSITIVE; NCT02308085) has just completed the recruitment of almost 500 patients to study whether temporary interruption is safe to allow pregnancy [13]. Jyoti et al. reported a young BC patient who discontinued endocrine therapy, and she became pregnant three times after diagnosis. Unfortunately, she developed locoregional recurrence 6 years after diagnosis [32]. In our report, the patient became pregnant within the first year of adjuvant ET. She then developed an ipsilateral chest wall recurrence 5 years after diagnosis. Until the results of the POSITIVE trial are out, for patients who are willing to interrupt ET to become pregnant, counselling on the recurrence risk, multidisciplinary decisions and close follow-up are required.

## Conclusion

We have reported the first case of pregnancy after synchronous BC–TC. Physicians need to be cognisant that thyroid and breast cancers might occur in the same patient metachronal or synchronal. Therefore, breast and thyroid examination should always be carried out together during diagnosis and follow-up. Moreover, they should be aware of the feasibility of pregnancy in this group of patients.

To avoid an unexpected pregnancy, all premenopausal women should be counselled on the use of barrier contraceptive methods before initiating endocrine therapy. In case an unplanned pregnancy occurs, tamoxifen interruption is advised.

Since an increasing number of breast cancer survivors under adjuvant endocrine therapy are willing to become pregnant, and only retrospective data support the currently available evidence, the timing and safety of interrupting endocrine therapy should be decided case by case, discussed with the patient and documented in detail. The awaited results of the POSITIVE trial will offer guidance on the safety of endocrine therapy interruption for one to become pregnant.

## List of abbreviations

5-FU: Fluorouracil
PTEN: Phosphatase and tensin homolog gene
PI3K: Phosphatidylinositol 3-kinase pathway

## Conflicts of interest

The authors declare that they have no conflicts of interest.

## Funding

No funding was received for this work.

## Disclosure

This case report was presented at the European School of Oncology case discussions.

## Authors’ contributions

Abeid M Athman Omar, Amany Abdelbarry and Rasha O. Elsaka substantially contributed to the conception, drafting and revising the work and approved the final manuscript to be published. All authors agree to be accountable for all aspects of the work in ensuring the accuracy and integrity of the work.

## Patient consent

Consent was obtained from the patient prior to the preparation of this case report.

## Figures and Tables

**Figure.**
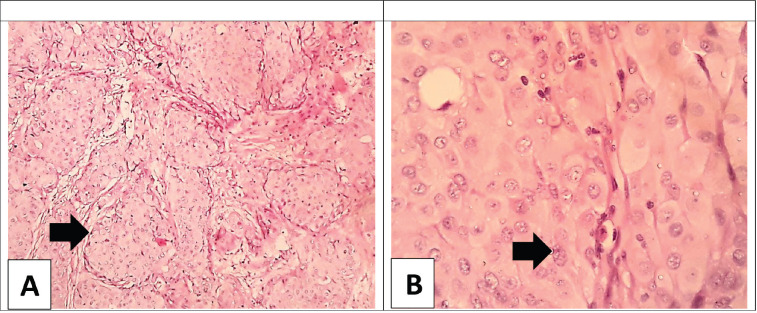
(A): Low power view of the breast infiltrating ductal carcinoma; NOS showing cords of large malignant cells (arrow) separated by scant stroma; no papillae are detected (H&E × 100). (B): Higher power view of the breast carcinoma; the malignant cells are large with vesicular pleomorphic nuclei, coarse chromatin, with no classic nuclear features of papillary carcinoma; (arrow) the cytoplasm is ample and eosinophilic (H&E × 400).
